# Letter from Samuel J. Jones, M.D.

**Published:** 1868-09-15

**Authors:** Samuel J. Jones

**Affiliations:** London, England


### *FOREIGN CORRESPONDENCE.*

London, England, *August* 12*th*, 1868.

My Dear Doctor: I have endeavored quite a number of times, since I last saw you, to find time to write you a few lines, and some for the Journal, in accordance with your suggestions before I left Chicago, but I have been moving so constantly that I have found it almost impossible. I landed at Queenstown, Ireland, on the 16th of June, and visited Cork and the Killarney Lakes, after which I went to Dublin, where I spent about two weeks most delightfully. I met Stokes, Sr. and Jr., Wilde, Hamilton, Banks, Beatty, Mapother and others. I was shown the professional, as well as other sights of the city—was wined and dined by them, both in town and at their country places, and was most hospitably treated every where by them. I attended the commencement exercises of the Dublin University (Trinity College), and visited most of the medical schools and hospitals, of which I will try to write you more hereafter. From Dublin I went to Belfast and the Giant's Causeway, and then to Glasgow, having been "ticketed" on by my Dublin friends and consigned to Dr. Gardiner of the Glasgow University, Dr. Cowan and Dr. Buchanan. I had a very pleasant visit of a few days there, and saw much of professional interest. From there I went to Oban, on the west coast of Scotland, and crossed through the Highlands, and visited Lochs Lomond and Katrine, the Trossachs, and passed on through Stirling to Edinburgh, where I

met Sir James Simpson, Syme, Hughes, Bennett, Watson, and quite a number of the other notabilities. I went with Bennett to the Edinburgh University, and saw his dogs, on which he was making his experiments, which seem to say that our ideas of the action of *Mercury* have not been correct. I spent about two weeks there most agreeably, and just missed Dr. Freer and Dr. Chas. G. Smith, who had been there a few days before. From there I returned to Glasgow, and spent a few days more, and then went to Liverpool, and spent a couple of days in looking up the sanitary matters of that city, which are admirably conducted, and their system of public baths and wash-houses is a credit to any city. I have examined that matter closely in all the cities I have visited, and Liverpool compares most favorably so far as I have seen. From there I went to Chester, to see a fair specimen of an old walled city. From there I came to London, and in the three weeks I spent here, I met Sir Wm. Ferguson, Erichsen, Bowman, Critchett, Solly, Spencer, Wells, Hutchinson, Sibson, Lawson, and saw most of them operate. I have visited Guy's, St. Bartholomew's, London, Charing Cross, Royal Ophthalmic, Westminster Ophthalmic, Southwark Ophthalmic, Royal Orthopædic, and quite a number of other hospitals, most of which have colleges, as you know, connected with them. I have made notes of most of the matters of interest, and if I can find time I will try soon to put some of them in shape for your journal. After making quite good use of the time I spent here, I went to Oxford to attend the meeting of the British Medical Association, to which I was a delegate, and which met on the 4th inst. Of this I will send you, herewith, a brief account, and I have sent, to Dr. Lyman, papers containing an account of most of the general order of the proceedings, and some of the works. I desire to preserve them, but if you desire to consult any of them he will place them at your disposal for the time being.

I start for Paris to-morrow, where I shall remain a short time, and be in Heidelberg on the 3rd, 4th and 5th of September at the Congress of the Ophthalmia men of Europe, where, it is expected, Graefe, Donders, Stellwag, Arlt, Critchett and others will be. After that I expect to go to Berlin, Dresden and Vienna, and if, in the meantime, I can catch a few spare hours, I will try to place the result of them at your disposal. I should be glad to hear from you at any time. Address me—Care of Norton & Co., Bankers, 14 Rue Auber, Paris. They will forward any letters for me. Hoping to see you in a few months, and with kind remembrances to all friends, I remain very truly yours,

SAMUEL J. JONES.

The thirty-sixth annual meeting of the British Medical Association occurred at Oxford, on the 4th of August, and was organized by Professor Stokes, of Trinity College, Dublin, the President of the Association for the past year.

In accordance with the custom of the Association, after organizing the meeting, the President delivers a valedictory address and resigns the chair to the President elect, who delivers an inaugural address, which duty this year devolved upon Professor Ackland of the University of Oxford. This generally completes the work of the first day. The next day generally begins the real work of the meeting. The arrangements for the general meetings of the entire Association, and for the different sections of medicine, surgery, obstetrics, etc., are made, and assignment is made to each of its proper share of work. The delegates from other Medical Associations are then admitted. The American Medical Association was this year represented by Dr. S. D. Gross and Dr. H. E. Goodman, of Philadelphia. Dr. Fordyce Barker, of New York, and Dr. S. J. Jones, of Chicago, as delegates to the Oxford Meeting.

During the general meetings of the Association addresses were delivered by Professor Rolleston, of the University of Oxford, on Physiology; by Dr. Gull, of London, on Medicine, its present status, etc.; by Professor Haughton of Trinity College, Dublin, "Sources of vital and mechanical force derived from food, and its influence on medical practice," and by Professor Bennett, of the Edinburgh University, on the action of mercury. The latter address was accompanied by tables showing the result of a series of experiments (upon dogs, in which biliary fistula had been made, under Professor Bennett's direction), which are adduced as evidence that mercury does not increase the secretion of bile. The recognized position of the author of the address, the apparent care with which the experiments were conducted, and the importance of the questions entitle the subject to consideration, and lead us to hope that something satisfactory may result from the experiments to be continued.

The report of the Joint Committee of the British Medical and Social Science Associations on Public Medicine, appointed last year, was presented in the general meeting, and various subjects bearing upon the advancement of the medical profession, and regarding sanitary science were considered. In medical education, the same difficulties are experienced, and the same questions agitate the teachers, and interest the profession at large, in Great Britain, that have received so much attention in the United States, as to how the standard shall be elevated, and the proper protection secured for both practitioner and the public. Much is hoped for here from the establishment of the British Council of Medical Education, the only legal Medical Association of Great Britain.

The President's soiree was given at the Museum of the University, and afforded all an opportunity of examining that, and the "Annual Museum" of the Medical Association, which was inaugurated this year, and opened for the session in the building of the University Museum. It is a beginning of what promises to be an interesting feature of the annual meetings, and is one worthy of the consideration of the American Medical Association. The design is to show the advancement made in the profession from year to year by annually collecting together during the session, wherever it may occur, of new surgical instruments, interesting pathological specimens, valuable new medical works, etc., etc. Prominent among the latter, this year, were the works issued from the Surgeon General's office of the U. S. Army, and the flattering and frequent allusions made during the meeting to the American contributions to medicine and surgery were very gratifying. Before adjournment, the appointment of a deputation to attend the next meeting of the American Medical Association was authorized—their first delegates to that association.

The work of the sections is arranged much in the same manner as that of the American Association, the organization of the two associations being almost identical. A printed daily bulletin is issued here, stating the work of the different sections for the day, in addition to matters of general interest, which, if imitated by our own association, would prove a convenience to the members.

In the surgical section, much of interest was presented, though comparatively little that was new. The subject of torsion as a hæmostatic means received considerable attention. The results of quite extended experiments were given, and seemed to indicate that in medium and small size arteries it is reliable and valuable, but could scarcely be considered so in large vessels. Acupressure was not specially considered. On the subject of the value of carbolic acid in surgery, much difference of opinion was manifested as to the range of its applicability.

One of the most ingenious apparatuses presented at the meeting was by Dr. Adams, of Maidstone, England. It is an instrument which he has devised for measuring the field of vision, which seems to be very reliable, and if so, will prove a valuable aid to ophthalmic surgeons, in supplying what has long been needed. It consists of a glass hemisphere, supported on an iron rod, with a slide to accommodate it to the height of the patient's eye. The eye of the surgeon is opposite that of the patient, who can thus see if the patient changes his eye in marking the field of vision, which is done by noting with a piece of soap on the glass hemisphere the limit of vision. The hemisphere is divided into meridians of longitude and parallells of latitude, and by having a similar diagram drawn on a small scale on paper, a convenient mode of recording the case for future use is afforded.

Many other matters of professional interest were presented in the different sections, but comparatively little that was new. Before adjourning, the annual elections took place, and Leeds, England, was decided upon for the next meeting, and in compliance with a rule of the Association, which requires that the President for the following year shall be from the district in which the meeting is to be held, Dr. Chadwick, of Leeds, was chosen as the presiding officer for the next term.

